# Iron-Doped Bimodal Mesoporous Silica Nanomaterials as Sorbents for Solid-Phase Extraction of Perfluoroalkyl Substances in Environmental Water Samples

**DOI:** 10.3390/nano12091441

**Published:** 2022-04-23

**Authors:** Enric Pellicer-Castell, Carolina Belenguer-Sapiña, Jamal El Haskouri, Pedro Amorós, José Manuel Herrero-Martínez, Adela R. Mauri-Aucejo

**Affiliations:** 1 Department of Analytical Chemistry, Faculty of Chemistry, Universitat de València, Dr. Moliner 50, 46100 Burjassot, Valencia, Spain; enric.pellicer@uv.es (E.P.-C.); carolina.belenguer@uv.es (C.B.-S.); jose.m.herrero@uv.es (J.M.H.-M.); 2 Institute of Material Science (ICMUV), Universitat de València, Catedrático José Beltrán 2, 46980 Paterna, Valencia, Spain; jamal.haskouri@uv.es (J.E.H.); pedro.amoros@uv.es (P.A.)

**Keywords:** mesoporous silica, perfluoroalkyl substances, water analysis, iron, solid-phase extraction, liquid chromatography

## Abstract

In this work, sorbets based on UVM-7 mesoporous silica doped with Fe were synthesized and applied to solid-phase extraction of perfluoroalkyl substances from environmental water samples. These emerging pollutants were then determined by liquid chromatography coupled with a mass spectrometry detector. Thus, Fe-UVM-7 mesoporous silica materials with different contents of iron, as well as different pore sizes (by using alkyltrimethilamonium bromide surfactants with different organic tail lengths) were synthesized, and their structure was confirmed for the first time by transmission electron microscopy, nitrogen adsorption–desorption, X-ray diffraction, and Raman spectroscopy. After comparison, Fe50-UVM-7-C_12_ was selected as the best material for analyte retention, and several extraction parameters were optimized regarding the loading and elution step. Once the method was developed and applied to real matrices, extraction efficiencies in the range of 61–110% were obtained for analytes with C_8_–C_14_ chain length, both perfluoroalkyl carboxylates, and perfluoroalkyl sulfonates. Likewise, limits of detection in the range of 3.0–8.1 ng L^−1^ were obtained for all target analytes. In the analysis of real well-water samples, no target compounds were detected. Spiked samples were analyzed in comparison to Oasis WAX cartridges, and statistically comparable results were achieved.

## 1. Introduction

Perfluoroalkyl and polyfluoroalkyl substances (PFASs) are a group of anthropogenic chemicals that have been widely used since 1950 for a wide range of industrial applications, such as polymer production or as surfactants [1]. Due to their persistence, ubiquity, and long-range transport potential, they have been detected in the environment, wildlife, and humans [2,3]. Despite their non-volatility and low water solubility, the most frequently detected PFASs are perfluoroalkyl carboxylates (PFCAs) and perfluoroalkyl sulfonates (PFSAs) [2,4], with increasing concern about their presence because of their bioaccumulation and adverse effects on humans [5,6]. In this sense, strong regulations are being applied to these compounds, mainly to perfluorooctanoic acid (PFOA) and perfluorooctane sulfonic acid (PFOS), which are included in Annex A and B of the list of the Stockholm Convention on Persistent Organic Pollutants, respectively, for their elimination or regulation [6,7,8,9]. Indeed, since they are considered emerging pollutants, the evaluation of these and other PFASs is currently ongoing, not only by the United Nations in the Stockholm Convention, but also by other organizations, such as the European Chemicals Agency, which has added several of these compounds to the Candidate List of Substances of Very High Concern [10,11,12].

Because of this, their monitoring and environmental vigilance have increased in recent decades, and many studies have been carried out to assess the presence of these pollutants in environmental matrices. In the case of water, several studies have reported the presence of significant concentrations of PFASs around the world. Although in European countries, lower concentrations of PFASs were detected [10,13,14], higher concentrations were found in Asian countries such as China, where concentrations up to several μg L^−1^ were reported [15,16,17]. Additionally, results showed that it was difficult for wastewater treatment plants to remove these compounds since significant concentrations of several PFASs were quantified in both influent and effluent channels from plants around the world [15]. Indeed, Lorenzo et al. [6] found maximum concentrations in the range of 20–60 ng L^−1^ in Valencia’s wetland (Spain), with these concentrations reaching 100 ng L^−1^ in the case of wastewater. Moreover, the transport of these compounds has also been studied, being an important concern due to their long-range transport [18,19,20].

These results have led to the development of a wide variety of methods for PFASs’ preconcentration, enrichment, and determination in environmental samples. Even though some groups of PFASs, mainly the fluorotelomers, can be determined by gas chromatography (GC) [2,21], in some cases of previous derivatization [22], the most commonly employed technique for the quantification of these analytes is liquid chromatography (LC) coupled with a mass spectrometry detector (MS) [23]. In this sense, although high-resolution liquid chromatography (HPLC) has been widely used for separation, the appearance of more sophisticated techniques has caused the migration to ultra-high-resolution liquid chromatography (UHPLC) [23,24]. However, and despite the great sensitivity of these techniques, the low concentrations generally present in some samples make the preconcentration step mandatory for environmental analysis. For this purpose, in the case of water samples, the most used technique is solid-phase extraction (SPE), although some studies using liquid–liquid extraction (LLE) have been reported [25,26]. Likewise, other miniaturized techniques have also been proposed for PFASs’ enrichment from water samples, such as solid-phase microextraction (SPME) with fibers, or dispersive liquid–liquid microextraction (DLLME), although a significantly lower volume of water can be treated in these cases, thus entailing poor enrichment factors concerning environmental analysis [23]. SPE consists of the use of a solid phase that selectively retains the desired analytes, with the aim of cleaning up the matrix interferences and preconcentrating the target compounds through the later elution with a smaller solvent volume [27]. Hence, research on the design of new sorbent materials for the retention of organic pollutants from the environment has received much attention, not only for SPE purposes but also for remediation studies [28,29], where the sorbent selection is a crucial step in the SPE method’s design. Thus, several sorbents have been used for the retention and extraction of PFASs [23,26], including the classical C18 cartridges, but the most commonly used are the weak anionic exchange (WAX) cartridges, such as the Oasis WAX [4,10,30,31] or Strata-X AW [32,33,34,35], or the hydrophilic/lipophilic balanced Oasis HLB cartridges [36]. It should be noted that in all these cases, the interaction between the analyte and the sorbents is based on exchange or affinity reactions (hydro or lipophilic). Thus, although comparable recoveries have been reported for both of them, WAX cartridges are proven to be more efficient for short-chain PFASs, mainly in the case of ionic PFCAs and PFSAs, which are the most frequently used ones [26].

On the other hand, mesoporous silica materials have been used for the adsorption of several organic pollutants, such as PFASs from different matrices, including environmental samples [37,38]. These materials have received a great deal of attention since the appearance of the M41S solids developed by the Mobil Corporation, due to their high surface area, chemical and mechanical stability, and ease of controlling the particle size and morphology, with MCM-41 as the most studied solid [39,40,41]. In the same way, UVM-7 materials can be considered as a nanometric version of MCM-41, consisting of a continuous silica network constructed from aggregated small mesoporous nanoparticles, generating a non-ordered system of large macropores. This bimodal morphology is achieved through the introduction of surfactants in the synthesis procedure, based on the atrane route, allowing the control of the mesopore size and morphology depending on the used surfactant [42,43,44]. Moreover, their one-pot synthesis allows modifying the material through organic functionalization or the addition of metallic heteroelements to improve their selective properties. This functionalization can be achieved by simply adding the corresponding metallic precursor to the initial solution, leading to an M-UVM-7 structure [44,45]. These features, jointly with the cheap synthesis procedure and the reversible interactions established with analytes, have led to several SPE-based applications of UVM-7 materials in environmental analysis, such as the determination of metal ions [46] or the extraction of organophosphorus and organochlorine compounds from water [45,47]. However, it should be noted that the simultaneous modification of the mesopore size and the introduction of metallic sites along the mesopore walls have not been yet studied and are explored here for the first time. Thus, the presence of iron has proved to improve the effectiveness of the sorption of PFASs in some studies focused on pollution removal; thus, the addition of this metal is a possible strategy to enhance the selectivity of SPE sorbents for the extraction of these compounds [48,49].

The aim of this work is to evaluate Fe-UVM-7 materials with different porosities and Fe contents for PFASs’ selective retention in order to develop an SPE method for their preconcentration and determination in water samples. The later instrumental detection of the analytes is carried out by UHPLC-MS/MS. Once the method features are assessed, the protocol is applied to real water samples in comparison with commercial WAX cartridges, commonly used for PFASs’ extraction [10].

## 2. Materials and Methods

### 2.1. Chemicals and Reagents

A multicomponent solution of PFASs in methanol was purchased from Wellington Laboratories (Southgate, ON, Canada) containing 2000 μg L^−1^ of perfluoro-n-butanoic acid (PFBA), perfluoro-n-pentanoic acid (PFPA), perfluoro-n-hexanoic acid (PFHxA), perfluoro-n-heptanoic acid (PFHpA), perfluoro-n-octanoic acid (PFOA), perfluoro-n-nonanoic acid (PFNA), perfluoro-n-decanoic acid (PFDA), perfluoro-n-undecanoic acid (PFUnDA), perfluoro-n-dodecanoic acid (PFDoDA), perfluoro-n-tridecanoic acid (PFTrDA), perfluoro-n-tetradecanoic acid (PFTeDA), perfluoro-1-butanesulfonic acid (PFBS), perfluoro-1-hexanesulfonic acid (PFHxS), perfluoro-1-octanesulfonic acid (PFOS), and perfluoro-1-decaesulfonic acid (PFDS). A concentrated solution of perfluoro-n-[^13^C_8_]octanoic acid (^13^C_8_-PFOA) of 50 μg mL^−1^ in methanol was also purchased from Wellington Laboratories and used as an internal standard. These stock solutions were stored, refrigerated in the darkness, and diluted with methanol for their analysis.

During optimization and sample analysis, HPLC-grade methanol (MeOH) was used from Panreac AppliChem (Darmstadt, Germany), as well as ethyl acetate from Lab-Scan Analytical Sciences (Gliwice, Poland), dichloromethane (DCM) from VWR chemicals (Radnor, PA, USA), and acetonitrile (ACN) from Labkem (Barcelona, Spain). In addition, acetic acid from Sigma-Aldrich (St. Louis, MO, USA), NaOH from Scharlau (Barcelona, Spain), and NH_4_F from Merck (Darmstadt, Germany) were also employed. Ultrapure water was obtained from an Adrona (Riga, Latvia) purification system. For sample analysis, Oasis WAX cartridges containing 150 mg of sorbent were also used from Waters (Milford, MA, USA).

Throughout the synthesis of the iron-doped silica nanomaterials, ethanol from VWR Chemicals, tetraethyl orthosilicate (TEOS), and triethanolamine (TEA) from Fluka (Buchs, Switzerland) were used, as well as FeCl_2_·4H_2_O and the surfactants cetyltrimethylammonium bromide (C_16_TAB), dodecyltrimethylammonium bromide (C_12_TAB), and decyltrimethylammonium bromide (C_10_TAB) from Sigma-Aldrich. During the material preparation, all reagents used were of reagent grade.

### 2.2. Instrumentation

All solids were analyzed for Fe and Si content through energy-dispersive X-ray spectroscopy (EDX) by using a Philips XL30 ESEM (Amsterdam, The Netherlands) scanning electron microscope. X-ray powder diffraction patterns (XRD) were recorded on a Bruker D8 Advance diffractometer (Billerica, MA, USA) equipped with a monochromatic CuKα source operating at 40 kV and 40 mA. Transmission electron microscopy study (TEM) was carried out with a JEOL JEM-1010 instrument (Tokyo, Japan) operating at 100 kV and equipped with a CCD camera. Surface area, pore size, and volume values were calculated from nitrogen adsorption–desorption isotherms (−196 °C) recorded on a Micromeritics (Norcross, GA, USA) ASAP-2020 automated analyzer. Calcined samples were degassed for 5 h at 120 °C and 10^−6^ Torr prior to analysis. Surface areas were estimated according to the BET model, and pore size dimensions and pore volumes were calculated by using the BJH method from the absorption branch of the isotherms. Additionally, pore volumes were also estimated through the α_S_ method. Raman spectra were recorded by using Horiba-MTB Xplora equipment (Kyoto, Japan), using a 785 nm laser excitation source.

SPE experiences were carried out under vacuum by using a VacElut 20 connected to a vacuum pump. Water samples were filtered before their analysis using polyamide 0.45 μm filters from Sartorius Stedim Biotech (Goettingen, Germany).

The instrumental determination of PFASs was carried out with an ExionLC AD liquid chromatograph coupled to a triple quadrupole QTRAP 6500+ mass spectrometry detector from Sciex (Framingham, MA, USA). The separation of the analytes was achieved with a Kinetex C18 column (100 Å × 2.1 mm × 1.7 μm) from Phenomenex (Torrance, CA, USA), using a mobile phase composed of water and methanol containing 2.5 mM of NH_4_F in both cases as mobile phase modifier to improve the ionization efficiency in negative ionization mode [50]. The proportion of both solvents and the programmed gradient is shown in Table S1 (flow 0.3 mL min^−1^). Instrumental parameters of the MS/MS detector, including selected transitions for each analyte, are displayed in Table S2. The quantification of all studied analytes was properly carried out in the described conditions (Figure S1).

### 2.3. Synthesis of Silica Nanomaterials Doped with Fe

Hierarchically ordered mesoporous silicas were synthesized through a “one-pot” procedure previously described [42], denoted as the atrane route. In all cases, when the C_16_TAB surfactant was used, the molar ratio 2 − n Si: n Fe: 7 TEA: 0.5 C_16_TAB: 180 H_2_O was maintained to synthesize solids with (2 − n)/n = X = 100, 50, 25, and 10. Depending on the iron content, the resulting samples were labeled as FeX-UVM-7-C_16_. Thus, in a typical synthesis (Fe50-UVM-7-C_16_), 23 mL of TEA was mixed with 11 mL of TEOS and 0.19 g of FeCl_2_·4H_2_O and heated to 140 °C until reaching a homogeneous solution. Then, the solution was let cool until 120 °C, and 4.68 g of C_16_TAB surfactant was added under stirring. Finally, at 85 °C, 80 mL of ultrapure water was added, and the mixture was aged overnight under vigorous stirring at room temperature. The resulting solid was collected by vacuum filtration and gently washed with water and ethanol. After air-drying, the solid was dried in the oven at 80 °C. The final material was obtained after removing the surfactant by calcination at 550 °C for 6 h.

Moreover, in order to modify the mesopore size, related surfactants with shorter tail lengths were used, namely C_12_TAB and C_10_TAB. Thus, the Fe50-UVM-7-C_12_ and Fe50-UVM-7-C_10_ materials were obtained following the previously described synthesis for C_16_TAB. The previously molar ratio of the reagents was preserved with the exception of the surfactant value: 1.96 Si: 0.04 Fe: 7 TEA: m C_y_TAB: 180 H_2_O (m = 4.2 and 16.7 for C_12_TAB and C_10_TAB, respectively).

### 2.4. SPE Optimization, Recommended Procedure, and Sample Analysis

For all SPE experiments, cartridges were prepared by packing the desired amount of silica solid phase (300 mg of Fe50-UVM-7-C_12_ for the recommended procedure) between two polyethylene frits. In all cases, cartridges were conditioned with 5 mL of methanol followed by 5 mL of ultrapure water. After sample loading, cartridges were air-dried for 30 min. Finally, and following the optimized protocol, retained analytes were eluted with 6 mL of methanol. Then, the extracts were preconcentrated by evaporation under a N_2_ stream at 60 °C. After evaporation until 250 μL, the final extract was diluted with 175 μL of methanol and 75 μL of NH_4_F 8.3 mM prior to injection in the UHPLC-MS/MS system (final volume of 500 μL).

Water samples were obtained from the irrigation system from several points in the Valencia region. All samples were filtered through a 0.45 μm filter in order to remove any particulate matter and stored at 4 °C until analysis. Before SPE, all samples were adjusted to pH 4.6 with acetate buffer. The samples were analyzed through the recommended described protocol. In some cases, samples were spiked with PFASs standard solution (150 ng L^−1^). Spiked samples were also analyzed by a reference method using commercial Oasis WAX cartridges (150 mg). In these cases, cartridges were conditioned with 5 mL of NH_4_OH 0.1% in methanol, followed by 5 mL of methanol and 5 mL of ultrapure water. The elution procedure was also modified since 3 mL of methanol followed by 3 mL of NH_4_OH 0.1% in methanol were used in this case. The subsequent evaporation and concentration steps were carried out as described.

### 2.5. Analytical Figures of Merit

The precision, as well as the extraction efficiency of the proposed method, were assessed by analyzing replicates of 100 mL of a spiked real irrigation matrix (150 ng L^−1^ of each analyte) through the recommended procedure. In the case of intra-day precision, three replicates were carried out within a day, while for the inter-day precision, three series of three independent extractions were considered. The sensitivity of the method was also evaluated with the estimation of the limit of detection (LOD) and limit of quantification (LOQ), following the IUPAC recommendations, with a 95% confidence level [51]. The linearity was also studied, considering the LOQ as the lower limit of the linear range.

## 3. Results and Discussion

### 3.1. Synthesis and Characterization of Fe-Containing Silica Nanomaterials

The preparative strategy here used to include Fe atoms along the silica mesopore walls (the “atrane route”) lies in using mixtures of atrane-type complexes as hydrolytic precursors [44], where the relative inertness of the atrane species helps to orchestrate the hydrolytic rates involving different inorganic moieties. This, in turn, facilitates the harmonization of the subsequent self-assembling processes between the inorganic oligomers and the surfactant micelles. Usually, this strategy leads to a remarkable dispersion of the heteroelement, and the resulting Fe-UVM-7 solids do not show phase segregation at the micrometric scale (see below). On the other hand, the use of surfactants with variable tail lengths to modulate the mesopore size is a classical strategy used in the case of MCM-41 and related silicas [42], adapted in this case to the Fe-UVM-7 silicas. Therefore, to maintain the nanometric size of the UVM-7 primary particles, it is mandatory to significantly increase the amount of surfactant since its critical micelle concentration (cmc) values increase when decreasing the length of the surfactant tail [52]. In this way, the synthesis strategy guarantees the presence of similar and adequate concentrations of micelles in a solution for all surfactants used, and a very rapid nucleation process of UVM-7-type nanoparticles takes place, followed by limited growth.

Firstly, EDX was used to assess the stoichiometry and chemical homogeneity of the adsorbents. EDX data show that all reported samples are chemically homogeneous at the scale spot area (ca. 1 µm). Regardless of the nominal Si/Fe ratio or the surfactant used, the real Fe content in the final materials (Table 1) is higher than expected, considering the Si/Fe introduced in the initial stock solution. That is, Si/Fe molar ratios determined by EDX (hereinafter real values) are smaller than the stoichiometric values added in the synthesis (hereinafter nominal values). In the case of the solids synthesized with the C_16_TAB surfactant, an approximately stable increase in the range of 30–40% is observed. It is also noted that for an identical nominal molar ratio of Si/Fe = 50, when the size of the surfactant decreases, the enrichment in Fe is somewhat higher: 52 and 62% for the solids prepared with C_12_TAB and C_10_TAB, respectively. This fact indicates the preferential incorporation of Fe into the final silica network due to the iron oxide insolubility when compared to that of silica. If we consider that the materials can be described as mixtures of SiO_2_ and Fe_2_O_3_ oxides or Fe(OH)_3_, it is well known that the solubility of SiO_2_ is much greater than that of Fe_2_O_3_ (insoluble) or Fe(OH)_3_ (K_ps_ = 2.79 × 10^−39^) [53,54]. Thus, the Fe enrichment can be assigned to a partial silica dissolution (120 g L^−1^ in water) [55].

**Figure 1:**
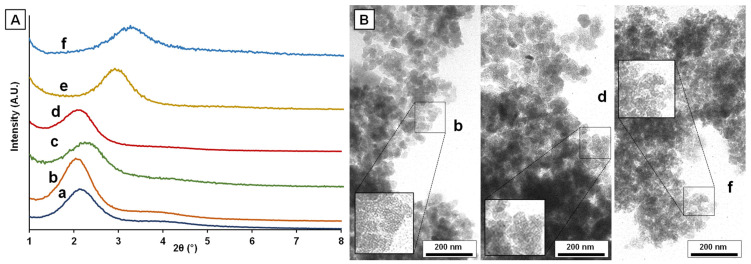
(**A**) Low-angle XRD patterns and (**B**) selected TEM images of the synthesized materials: (a) Fe100-UVM-7-C_16_, (b) Fe50-UVM-7-C_16_, (c) Fe25-UVM-7-C_16_, (d) Fe10-UVM-7-C_16_, (e) Fe50-UVM-7-C_12_, and (f) Fe50-UVM-7-C_10_. The images shown in the insets correspond to ×2 magnification.

**Figure 2:**
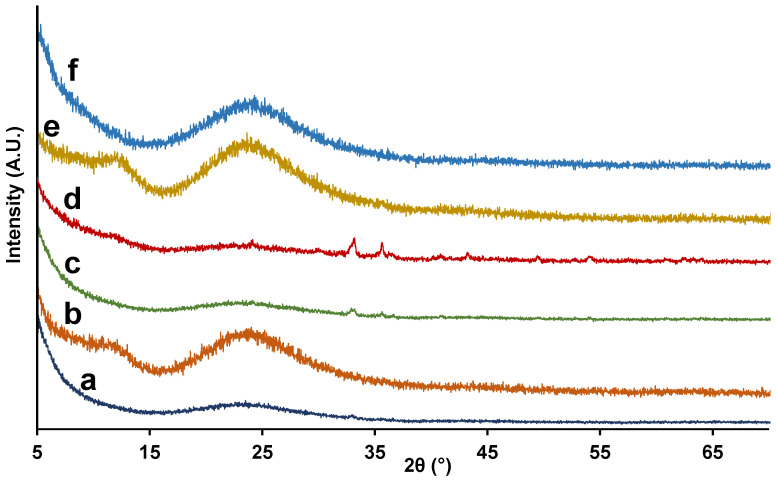
High-angle XRD patterns of the synthesized materials: (a) Fe100-UVM-7-C_16_, (b) Fe50-UVM-7-C_16_, (c) Fe25-UVM-7-C_16_, (d) Fe10-UVM-7-C_16_, (e) Fe50-UVM-7-C_12_, and (f) Fe50-UVM-7-C_10_.

All calcined solids display low-angle XRD patterns with one strong peak and one broad signal of relatively low intensity (Figure 1A), which can be associated with the (100) and the overlapped (110) and (200) reflections of an MCM-41-like hexagonal cell, respectively [39,40,41]. These patterns are characteristic of hexagonal disordered mesoporous UVM-7 materials, and they only inform us about the existence of the intraparticle mesoporous system. Regardless of the iron content, the position of the XRD signals is practically unchanged (see Table 1) for solids synthesized with the same surfactant (C_16_TAB). As expected, a gradual shift towards higher 2θ values (lower d_100_ spacing) occurs as the tail length of the surfactants decreases. Taking into account the high-angle XRD patterns of the materials (Figure 2), although the presence of small nanodomains of iron oxide (Fe_2_O_3_) is not completely discarded, it is expected to be negligible in comparison to the amount of iron introduced, thus entailing a homogeneous dispersion of the heteroelement in almost all the samples as molecular species or small clusters of sizes below the detection limit of XRD (ca. <4 nm) [56,57]. However, in the case of the Fe10-UVM-7-C_16_ sample, where the highest content of Fe was introduced, the low-intensity peaks associated with Fe_2_O_3_ nanodomains are clearly observed, which indicates the presence of iron oxide domains to a greater extent and, subsequently, a worse dispersion of the metal. This observation is also supported by the Raman spectrum of this solid (Figure S2), which shows the presence of hematite-like nanodomains in this solid, while these signals are practically negligible in the other measured solids.

Likewise, TEM images (Figure 1B) show that regardless of the Fe content or the surfactant used, the nanoparticulated bimodal porous structure typical of UVM-7 silicas is preserved. All materials present a continuous nanometric organization built from aggregates of small nanoparticles. The white spots observed inside these nanoparticles (see the insets in Figure 1B) are indicative of the existence of mesopores (as expected, taking into account the use of micelles as template agents). On the other hand, the interparticle voids generate a hierarchic non-ordered system of large pores.

**Figure 3:**
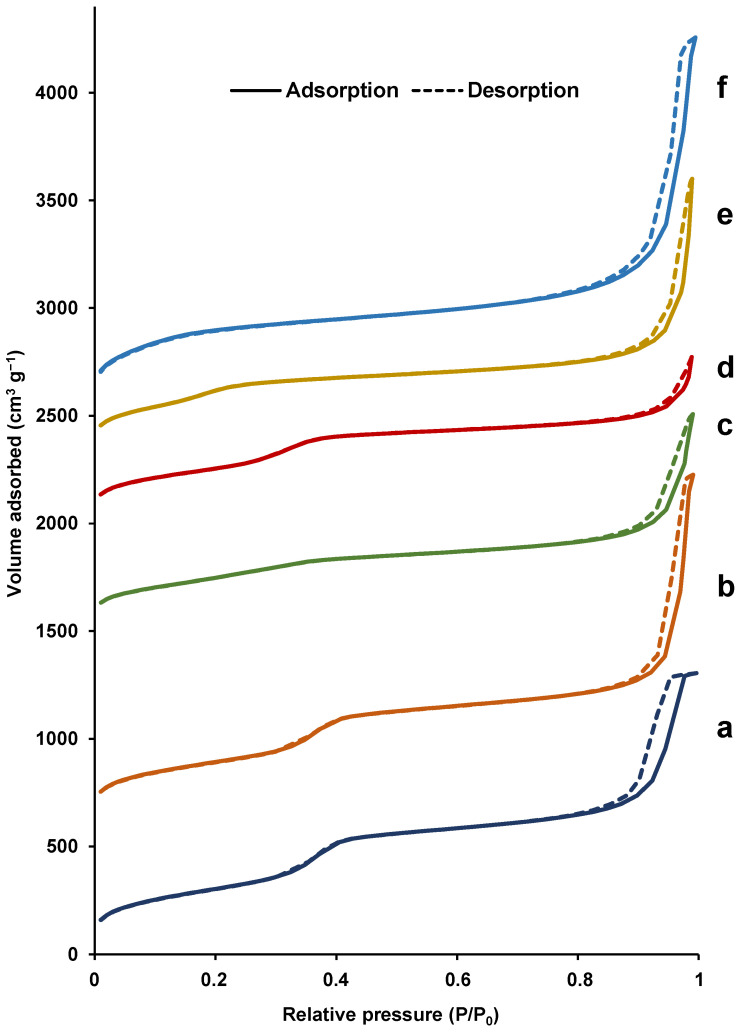
N_2_ adsorption–desorption isotherms of the synthesized materials: (a) Fe100-UVM-7-C_16_, (b) Fe50-UVM-7-C_16_, (c) Fe25-UVM-7-C_16_, (d) Fe10-UVM-7-C_16_, (e) Fe50-UVM-7-C_12_, and (f) Fe50-UVM-7-C_10_.

The bimodal pore system was unambiguously confirmed by N_2_ adsorption–desorption isotherms (Figure 3). In all cases, the solids show typical curves of UVM-7 silicas, with two well-defined adsorption steps characteristic of UVM-7 materials. The first, at intermediate partial pressures (0.1 < P/P_0_ < 0.4), is due to capillary condensation of N_2_ inside the intrananoparticle mesopores. The second step, at high relative pressure (P/P_0_ > 0.8), corresponds to the filling of the large interparticle pores. All solids show very high BET surface area values, greater than 1000 m^2^ g^−1^, except for solids with higher iron content, in which the area is slightly lower (Table 1). The interparticle pore shows a moderate heterogeneity in sizes and volumes already observed in other UVM-7 type materials. Thus, the mean size is 37.5 ± 9 nm, and the volumes are always greater than 1.20 cm^3^ g^−1^, with the exception of the two Fe-richest solids, for which the interparticle BJH volume gradually decreases as the Si/Fe ratio decreases. In any case, taking into account the size and linear shape of perfluoroalkyl substances, the interparticle pore system does not represent any barrier to their easy diffusion. More significant differences are seen in intraparticle porosity. The size of the mesopore, as well as the BJH pore volume, is affected by both the incorporation of Fe as well as by the size of the tail of the surfactant used. This decrease is lower in the case of materials synthesized from C_16_TAB (from ca. 2.8 to 2.5 nm by increasing the amount of Fe incorporated), and it is substantially more notable when C_12_TAB and C_10_TAB were used (2.26 and 2.01 nm, respectively). In addition, it should be noted that a good compromise between pore volumes estimated using BJH and α_S_ methods was obtained, with those calculated through the α_S_ method being slightly lower.

Hence, we have combined here, for the first time, with satisfactory results, the modification of UVM-7 materials with the simultaneous addition of metallic heteroelements and the use of short-chain alkyltrimethylammonium-type surfactants.

### 3.2. Solid-Phase Evaluation and Optimization of SPE Parameters

**Figure 4:**
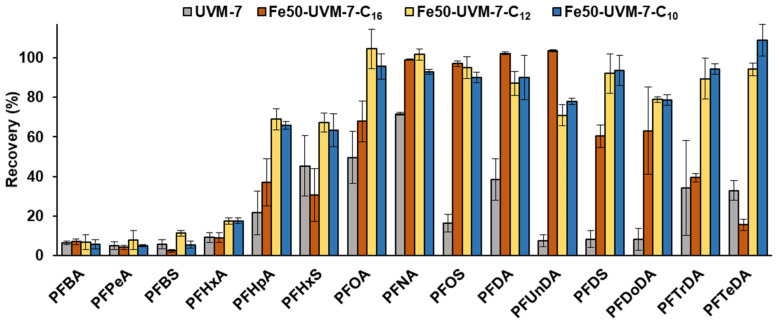
Effect of the sorbent (presence of Fe and pore size) on the recovery of PFASs. Conditions: 150 mg of sorbent, 10 mL of spiked ultrapure water (1 μg L^−1^), elution of 2 mL of methanol.

Firstly, UVM-7 materials containing several amounts of iron were tested for the adsorption of PFASs. For this purpose, extractions of 10 mL of spiked ultrapure water (1 μg L^−1^) were performed with cartridges containing 150 mg of each material. As can be seen in Figure 4, an important improvement was observed in most of the analyte recoveries when iron was introduced into the sorbent, in comparison with the blank UVM-7, as can be seen for the Fe50-UVM-7-C_16_ material, thus confirming that Fe centers play a beneficial role in the retention of fluorinated compounds. However, it should be noted that more iron introduction did not always imply greater recoveries since an important decrease in the recoveries was observed for the Fe10-UVM-7-C_16_ material. This behavior can be explained according to characterization observations since the presence of Fe_2_O_3_ nanodomains was observed in the high-angle XRD pattern of the Fe10-UVM-7-C_16_ sample and confirmed with Raman spectroscopy. Therefore, the distribution of Fe atoms along the internal surfaces of the mesopores can be expected to be more irregular. This fact might imply heterogeneity in the interaction between the sorbent surface and the analytes (depending on the distribution and nature of the Fe species). Because of that, the molar ratio Si/Fe = 50 was selected as the best option.

Even though the recoveries for some of the analytes (PFNA, PFOS, PFDA, and PFUnDA) were around 100%, the extraction performance for other analytes, mainly those too small or too large, was still unsatisfactory. This could be explained by the characterization data regarding the porous structure of the UVM-7 materials, whose mesopore size was not appropriate for these PFASs. For this reason, other materials with smaller pores were considered—thus using shorter surfactants in the synthesis process—in order to achieve smaller micelles and, consequently, smaller pore diameters. As shown in Figure 4, the assessment of these materials for PFASs retention showed a considerable increase in the retention of several analytes, such as PFHpA, PFOA, PFDS, PFTrDA, or PFTeDA, when Fe50-UVM-7-C_12_ was used, with recoveries above 60%—although these results were similar for Fe50-UVM-7-C_10_. Thus, Fe50-UVM-7-C_12_ was chosen as the best solid phase for the development of our method (since its synthesis is cheaper and easier due to the surfactant amount needed to keep the critical micellar concentration). In this sense, the use of C_12_TAB supposes a significant reduction in mesopore size (of almost 0.6 nm). In addition, the material does not show a significant presence of Fe_2_O_3_ nanodomains, which guarantees a homogeneous distribution of Fe along the surface of the mesopores. Both factors lead to the optimal capture of the analyte. Furthermore, it should be noted at this point that the adsorption mechanism differs from that operating for commercial adsorbents. Taking into account the linear nature of PFASs molecules (with a mean diameter much smaller than that of intraparticle mesopores) and the significant improvement in their retention when Fe is incorporated into the silica walls, our hypothesis about their retention is that it is based on the encapsulation of PFAS within the mesopores and the probable benefits from a certain interaction between the F atoms and some Fe centers distributed on the surfaces of the pore walls. However, unsatisfactory recoveries were obtained for the short-chain analytes, with recoveries below 20% for all tested sorbets. Because of that, this method cannot be applied for these short-chain PFASs, and the method was developed for its application to long-chain PFASs (C_8_–C_14_) extraction from water samples.

Once the solid phase was selected, the loading conditions were studied for selected analytes. First, three extractions of 10 mL of spiked ultrapure water (1.5 μg L^−1^) were performed at pHs 2.7, 4.6, and 6. In this case, no great differences were observed since all these pH values are in the range in which silica is stable [55], and the target analytes are expected to be in their anionic form in a wide range of pH due to their low pK_a_ values [58]. However, slightly better recoveries were obtained when the acetate buffer was used (pH 4.6), and this condition was selected for the method in order to work in a stable pH range and ensure the presence of all analytes in the anionic form. The loading ionic strength was also tested by adding several amounts of NaCl to the sample before the SPE. However, in this case, no significant variations were observed among extractions. This observation agrees with the behavior already observed in the case of UVM-7 materials for the retention of organic compounds from aqueous samples [45,47]. The elution step was also evaluated by testing several organic solvents for the selected PFASs elution from the cartridges. Similar extractions of spiked ultrapure water (1.5 μg L^−1^) were carried out as described with cartridges containing 150 mg of Fe50-UVM-7-C_12_ and using 3 mL of ethyl acetate, dichloromethane, acetonitrile, and methanol for the elution step. Obtained results showed that the worst recoveries were obtained with dichloromethane (below 40% in almost all cases), probably due to its lower polarity. Although better results were obtained for acetonitrile and ethyl acetate, the best recoveries were clearly achieved with methanol, in the range of 79–106%, since it is the most polar. Thus, methanol was the solvent selected. The possibility of concentrating the final extract by evaporation was also assessed. For this purpose, methanol solutions of the selected PFASs (2 μg L^−1^) were evaporated at 60 °C with either a vacuum chamber or an N_2_ stream. No important loss of the analytes was observed with either of the procedures, although better results (variations under 10%) were obtained with nitrogen evaporation, and this was the protocol selected for analyte preconcentration.

In order to assess the breakthrough volume, several sorbent amounts (between 150 and 300 mg) were tested for the treatment of different volumes of spiked water real matrices, up to 100 mL. In all cases, the total amount of analytes was maintained (15 ng). Obtained results showed that, with 150 mg cartridges, recoveries clearly decreased when the sample volume was increased from 10 to 100 mL. However, satisfactory recoveries were obtained for all selected analytes when 300 mg of Fe50-UVM-7-C_12_ was used, even with 100 mL of spiked water. Thus, these cartridges were selected for the method’s development, allowing us to treat up to 100 mL of water sample with no loss of the analytes.

### 3.3. Method Performance

The developed method was evaluated in terms of sensitivity, linearity, and precision. As shown in Table 2, good extraction efficiencies were finally obtained for target PFASs (C_8_–C_14_), with values above 61% for all of them and close to 100% in the case of PFOS or PFNA. It should be noted that these recoveries are comparable to other methods reported in the literature for PFASs’ extraction (Table 3), and better in some cases, which represents an important advantage for the quantitative extraction of analytes. It should also be mentioned that important differences between extraction efficiencies of different PFASs were detected not only in this study but also in the other methods described, and thus it is difficult to compare these recoveries. Additionally, in some cases, relative recoveries were reported, whilst absolute recoveries are considered in our case, and simpler studies are also reported in some cases with the evaluation of only one analyte. However, the lower recoveries for some analytes make it advisable to use a standard addition calibration, and this was considered for sample analysis henceforth. Additionally, as observed in the tables, these extraction efficiencies lead to enrichment factors (EF) in the range of 121–212, which is better than other methods where better recoveries were achieved due to the lower samples volume and the reduction in the preconcentration. In the same way, satisfactory repeatability was observed, with RSD values below 22% for intra-day precision, and in the range of 17–29% for inter-day precision, except in the case of PFOA, where lower precision between independent days was observed (38%).

The sensitivity of the method was also assessed in terms of LOD and LOQ, as well as linearity. As shown in Table 2, the developed method allows quantifying the target PFASs in concentrations above 25 ng L^−1^, with detection limits in the range of 3–8 ng L^−1^ and good linearity in the studied range of concentrations. This sensitivity is comparable with some of the methods summarized in Table 3, although in some cases where the used sample volume is higher, this sensitivity is not improved with the reported protocol. However, it should also be noticed that the methods for the estimation of LODs and LOQs are diverse in all reported works, which makes the sensitivity comparison less reliable.

Finally, the reusability of the cartridges containing the developed material was evaluated by carrying out several consecutive extractions, as previously described, using the same cartridge. In this case, after each use, cartridges were washed with 10 mL of methanol to ensure the elimination of any trace of the analytes and conditioned again as described. Results obtained showed that, after five uses of the same cartridge, recovery variations can be attributed to the dispersion of the method and not to a decrease in the extraction efficiency of the material. Thus, the developed cartridges were demonstrated to be reusable up to at least five times, which represents an important advantage against other disposable cartridges. Moreover, after these extractions, the preservation of the material structure was proved by XRD (Figure S3), thus confirming the stability of the developed material in the selected working conditions.

### 3.4. Water Analysis

Six water samples were collected from the irrigation system in the region of Valencia and analyzed through the developed method. However, no target PFASs were detected in any of the samples, with values < LOD in all cases. Because of that, two of these samples were spiked with the PFASs solution (150 ng L^−1^) and analyzed by the developed method, as well as the reference method using Oasis WAX cartridges. As can be seen in Table 4, comparable results were obtained by both methods, and values close to the theoretical values were achieved. The concordance between both methods was also confirmed with a t-student statistical comparison of means.

Hence, the developed method can be properly applied to the determination of selected PFASs (C_8_–C_14_) in real water samples, and thus it is an alternative for environmental analysis, with good analytical features for these analytes and featuring a cheap material for the extraction and preconcentration step.

## 4. Conclusions

Several nanomaterials were synthesized in this work for the extraction of PFASs from water samples. All materials were demonstrated to maintain the silica framework characteristic of UVM-7 materials, with the presence of both macro- and mesopores, despite the addition of iron to the structure. The iron incorporation was shown to be homogeneous in all samples except for the sample with the highest Fe content, where iron oxide nanodomains were observed. The porosity of the sorbents was also controlled and modified, thus decreasing the mesopore size with the use of short-tail surfactants. Hence, the possibility of the simultaneous introduction of iron into the UVM-7 structure with the pore modification was proved for the first time.

With Fe50-UVM-7-C_12_, an analytical method for the determination of PFASs in environmental water samples was developed, with satisfactory extraction efficiencies in the range of 61–110% for long-chain analytes (C_8_–C_14_). However, poor retentions were observed for short-chain PFASs with the developed material. Hence, the developed method presents analytical features that are generally comparable to or better than other similar methods reported in the literature, with good LODs in the range of 3.0–8.1 ng L^−1^, as well as good extraction efficiencies. The optimized protocol was also applied to the analysis of real water samples in comparison with a commercial sorbent. Although no target PFASs were detected in the analyzed samples, the analysis of spiked real samples, in comparison to commercial WAX cartridges, confirmed this method as an alternative for target PFASs’ determination in water samples.

## Figures and Tables

**Table 1:** Selected parameters of the synthesized mesoporous silica materials.

Material	Si/Fe (Real Molar Ratio)	Surface Area (m^2^ g^−1^)	d_100_ (nm)	Mesopore Size (nm)	Mesopore Volume (cm^3^ g^−1^)		Large Pore Size (nm)	Large Pore Volume (cm^3^ g^−1^)
Fe100-UVM-7-C_16_	63	1109	4.1	2.81	0.82 ^a^	0.77 ^b^	27.4	1.20
Fe50-UVM-7-C_16_	29	1067	4.2	2.83	0.77 ^a^	0.70 ^b^	44.4	1.75
Fe25-UVM-7-C_16_	14	907	4.2	2.58	0.45 ^a^	0.39 ^b^	36.4	1.05
Fe10-UVM-7-C_16_	6	931	4.2	2.55	0.57 ^a^	0.56 ^b^	34.9	0.55
Fe50-UVM-7-C_12_	24	1171	3.0	2.26	0.48 ^a^	0.44 ^b^	43.3	1.47
Fe50-UVM-7-C_10_	18	1046	2.7	2.01	0.28 ^a^	0.27 ^b^	34.7	1.28

^a^ Estimated using the BJH method. ^b^ Estimated using the α_S_ method.

**Table 2:** Analytical figures of merit of the developed SPE-UHPLC-MS/MS method for C_8_–C_14_ PFASs from water samples.

Compound	Linearity ^a^ (μg L^−1^)	Sensitivity (ng L^−1^)		Extraction Efficiency (%)	EF	Repeatability RSD (%)	
		LOD	LOQ			Intra-Day	Inter-Day
PFOA	2–50	4	12	66 ± 20	132	22	38
PFNA	4–50	7	22	110 ± 30	212	16	28
PFOS	2–50	3	9	110 ± 30	212	19	29
PFDA	5–50	8	24	77 ± 16	154	15	21
PFUnDA	4–50	7	21	73 ± 15	145	17	21
PFDS	2–50	3	11	67 ± 14	134	21	21
PFDoDA	4–50	6	18	75 ± 13	151	13	17
PFTrDA	5–50	8	25	68 ± 17	136	14	25
PFTeDA	2–50	3	11	61 ± 14	121	18	23

^a^ Referred to the final injection solution.

**Table 3:** Comparison of the proposed method with other reported SPE methods from the literature for the extraction of C_8_–C_14_ PFASs from water samples.

Sample	Sorbent	LOD (ng L^−1^)	LOQ (ng L^−1^)	Recovery (%)	RSD (%)	EF (EF_max_)	Solvents	Instrumental Determination	Ref.
Well water (100 mL)	Fe-UVM-7-C_12_ (300 mg)	3–8	9–25	61–110	14–29 (38) ^a^	121–212 (200)	11 mL MeOH	UPLC-MS/MS	This work
Wastewater (200 mL)	Oasis WAX (60 mg)	0.2–5.8	1.7–11	19–99	2–22	76–396 ^b^ (400)	8 mL MeOH	HPLC-MS/MS	[4]
Rainwater (200 mL)	Oasis WAX (150 mg)	- ^c^	0.02–0.1	97–132	6–15	24–66 ^b^ (25–50)	12 mL MeOH	LC-MS/MS	[30]
Surface and tap water (50 mL)	Oasis WAX (150 mg)	0.3–0.5	1.5–2.5	83–102 ^d^	0.7–6.2	104–127 ^b^ (125)	15 mL MeOH	LC-MS/MS	[31]
Surface, tap, and wastewater (250 mL^−1^ L)	Strata-X AW (200 mg) (+carbon clean-up)	0.3–3	- ^c^	38–104	4–33	380–1040 ^b^ (1000)	26 mL MeOH	LDTD-HRMS	[32]
River, ground, and drinking water (250 mL)	Strata-X AW (200 mg)	- ^c^	4–10	(15) ^a^ 49–103 ^d^	7–35 (180) ^a^	(375) ^a^ 1225–2575 ^b^ (2500)	15 mL MeOH 1.4 mL DCM 0.6 mL IPA	UHPLC-MS/MS	[33]
Surface and wastewater ^e^ (200 mL)	Strata-X AW (200 mg) (+carbon clean-up)	- ^c^	- ^c^	98–113	1.8–7.3	1960–2260 ^b^ (2000)	15 mL MTBE 3 mL MeOH	HPLC-MS/MS	[34]
River water (250 mL)	Strata (200 mg)	- ^c^	0.01–2	60–92 ^d^	10–18	600–920 ^b^ (1000)	12 mL MeOH	LC-MS/MS	[35]
Surface water ^e^ (10 mL)	Oasis HLB (225 mg)	0.1	0.5	61–83	3–10	6.1–8.3 ^b^ (10)	7 mL MeOH	LC-MS/MS	[36]
Wastewater (1 L)	Imidazolium-based IL (passive) (30 mg)	0.2–0.3	0.7–1	53.7–110	<13	- ^c^	6 mL MeOH	HPLC-MS/MS	[59]
Drinking water (35 mL)	Presep PFC-11 (60 mg)	- ^c^	5–25	83.2–112.4	0.1–0.4	58–79 ^b^ (70)	11.5 mL ACN	LC-MS/MS	[60]

^a^ Value in brackets refers to a value far out or range for only one analyte. ^b^ Not reported. Calculated from reported experimental data. ^c^ Not reported. ^d^ Values reported as relative recoveries. ^e^ Study developed only with C_8_ analytes (PFOA and PFOS). MTBE: methyl tert-butyl ether; IPA: isopropyl alcohol; LDTD: laser diode thermal desorption.

**Table 4:** Obtained concentrations (ng L^−1^) of target PFASs in the analyzed spiked water samples (spiking level 150 ng L^−1^).

Compound	M1S		M2S
	Fe50-UVM-7-C_12_	Oasis WAX	Fe50-UVM-7-C_12_
PFOA	165 ± 15	150 ± 9	152 ± 5
PFNA	152 ± 15	149 ± 15	150 ± 2
PFOS	161 ± 6	155 ± 15	147 ± 12
PFDA	152 ± 12	151 ± 15	147 ± 3
PFUnDA	147 ± 8	157 ± 10	148 ± 7
PFDS	160 ± 13	141 ± 5	140 ± 14
PFDoDA	138 ± 18	156 ± 8	150 ± 18
PFTrDA	130 ± 20	139 ± 6	138 ± 12
PFTeDA	153 ± 18	164 ± 3	152 ± 8
PFOA	165 ± 15	150 ± 9	152 ± 5

## Data Availability

Not applicable.

## Supplementary Materials

The following supporting information can be downloaded at: https://www.mdpi.com/article/10.3390/nano12091441/s1, Table S1: Mobile phase gradient used in UHPLC-MS/MS system for PFASs’ separation. Components: water (2.5 mM of NH_4_F) and methanol (2.5 mM of NH_4_F). Table S2: Instrumental parameters of the MS/MS detector in the determination of target PFASs. Figure S1: Example chromatogram of a standard solution of the studied PFASs, obtained with the described instrumental conditions: (1) PFBA, (2) PFPeA, (3) PFBS, (4) PFHxA, (5) PFHpA, (6) PFHxS, (7) PFOA, (8) ^13^C_8_-PFOA, (9) PFNA, (10) PFOS, (11) PFDA, (12) PFUnDA, (13) PFDS, (14) PFDoDA, (15) PFTrDA, and (16) PFTeDA. Figure S2: Raman spectra recorded for the synthesized materials. Figure S3: Low-angle XRD patterns of the Fe50-UVM-7-C_12_ material (a) before its use as a sorbent (reference) and (b) after being used five times for the extraction of PFASs from water samples.
