# Supplementary material for: Iron-Doped Bimodal Mesoporous Silica Nanomaterials as Sorbents for Solid-Phase Extraction of Perfluoroalkyl Substances in Environmental Water Samples
Source: Nanomaterials (Basel). 2022 Apr 23;12(9):1441. doi: 10.3390/nano12091441 (PMC9105103; doi:10.3390/nano12091441)
Supplement: Supplementary file 1 [file nanomaterials-12-01441-s001.zip › nanomaterials-1652465-Supplementary.pdf]

Supplementary Materials

Iron-Doped Bimodal Mesoporous Silica Nanomaterials as Sorbents for Solid-Phase Extraction of Perfluoroalkyl Substances in Environmental Water Samples

Enric Pellicer-Castell [1], Carolina Belenguer-Sapiña [1], Jamal El Haskouri [2], Pedro Amorós [2], José Manuel Herrero-Martínez [1] and Adela R. Mauri-Aucejo [1,*]

[1] Department of Analytical Chemistry, Faculty of Chemistry, Universitat de València, Dr. Moliner 50, 46100 Burjassot, Valencia, Spain; enric.pellicer@uv.es (E.P.-C.); carolina.belenguer@uv.es (C.B.-S.); jose.m.herrero@uv.es (J.M.H.-M.)
[2] Institute of Material Science (ICMUV), Universitat de València, Catedrático José Beltrán 2, 46980 Paterna, Valencia, Spain; jamal.haskouri@uv.es (J.E.H.); pedro.amoros@uv.es (P.A.)
* Correspondence: adela.mauri@uv.es

UHPLC-MS/MS instrumental conditions

Table S1. Mobile phase gradient used in UHPLC-MS/MS system for PFASs separation. Components: water (2.5 mM of NH_4F) and methanol (2.5 mM of NH_4F).

Time (min)	Methanol (%)
0	5
0.5	30
12	96
20	95
29	30

Table S2. Instrumental parameters of the MS/MS detector in the determination of target PFASs.

Compound	Retention time (min)	Quantifier transition				Qualifier transition			
		Q (m/z)	DP (V)	CE (V)	CXP (V)	q (m/z)	DP (V)	CE (V)	CXP (V)
**PFBA**	2.2	213 > 169	-80	-5	-10		-		
PFPeA	4.4	263 > 219	-80	-5	-10		-		
PFBS	4.9	299 > 99	-140	-38	-10	299 > 80	-140	-26	-10
PFHxA	6.1	313 > 269	-70	-5	-10	313 > 119	-70	-5	-10
PFHpA	7.3	363 > 319	-25	-5	-37	363 > 169	-25	-5	-37
PFHxS	7.5	399 > 99	-30	-78	-15	399 > 80	-30	-86	-9
PFOA	8.3	413 > 369	-32	-14	-31	413 > 169	-32	-14	-31
$^{13}C_8$-PFOA	8.3	417 > 372	-25	-14	-23	417 > 169	-25	-14	-23
PFNA	9.1	463 > 419	-30	-16	-17	463 > 219	-30	-16	-17
PFOS	9.1	499 > 99	-135	-108	-7	499 > 80	-135	-106	-9
PFDA	9.8	513 > 469	-60	-17	-49	513 > 269	-60	-17	-49
PFUnDA	10.3	563 > 519	-22	-14	-45	563 > 269	-22	-14	-45
PFDS	10.3	599 > 99	-80	-80	-10	599 > 80	-80	-80	-10
PFDoDA	10.8	613 > 569	-90	-5	-10	613 > 269	-90	-13	-10
PFTrDA	11.2	663 > 619	-100	-5	-10	663 > 169	-100	-24	-10
PFTeDA	11.6	713 > 669	-110	-5	-10	713 > 169	-110	-25	-10

*Entrance potential = -10 V
DP: Declustering potential
CE: Collision energy
CXP: Collision cell exit potential

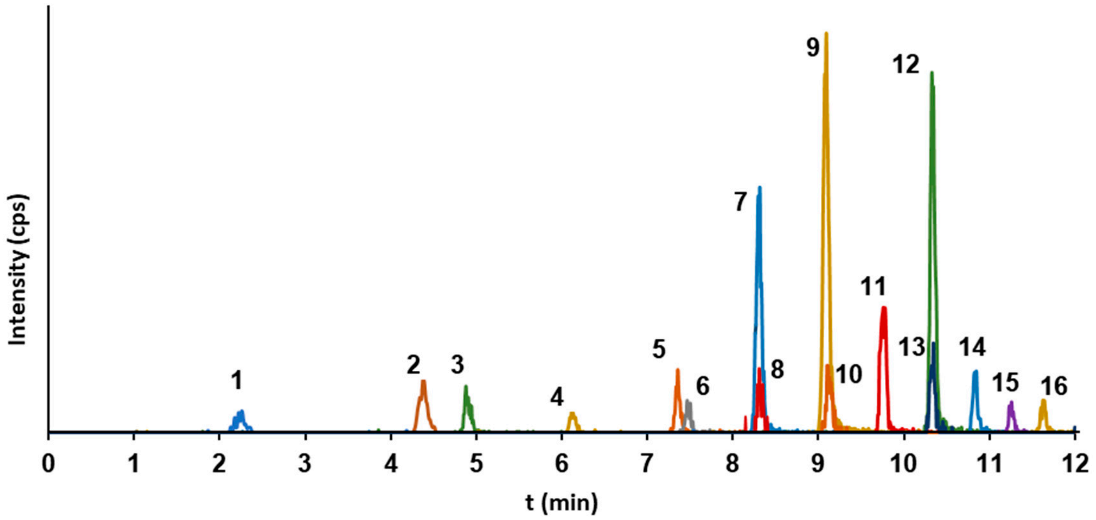

Figure S1. Example chromatogram of a standard solution of the studied PFASs', obtained with the described instrumental conditions: (1) PFBA, (2) PFPeA, (3) PFBS, (4) PFHxA, (5) PFHpA, (6) PFHxS, (7) PFOA, (8) $^{13}C_8$-PFOA, (9) PFNA, (10) PFOS, (11) PFDA, (12) PFUnDA, (13) PFDS, (14) PFDoDA, (15) PFTrDA, (16) PFTeDA.

Synthesis and characterization of Fe-containing silica materials

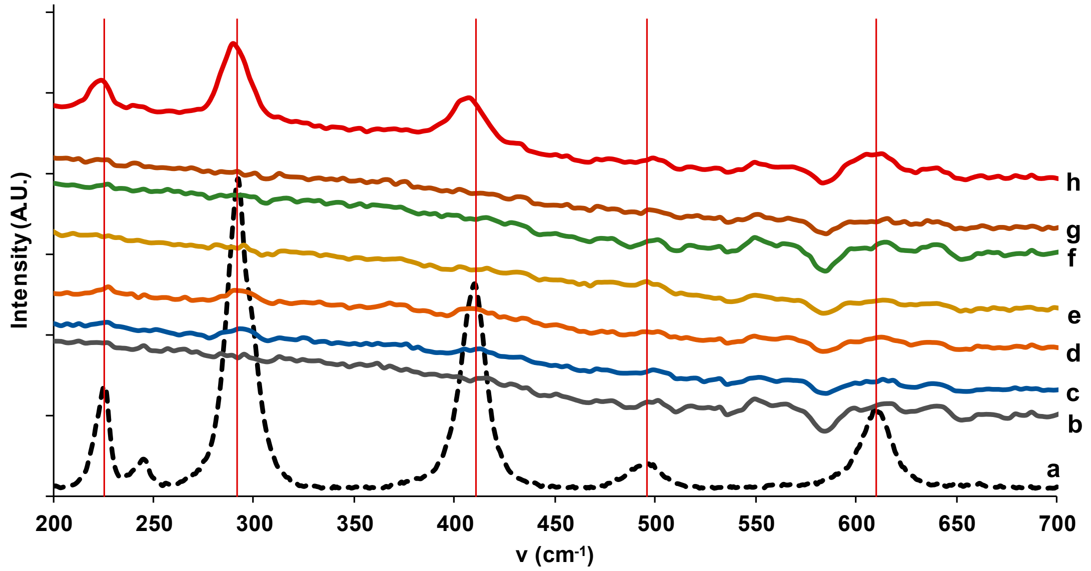

Figure S2. Raman spectra recorded for the synthesized materials: (a) Fe_2O_3 hematite reference, (b) UVM-7, (c) Fe100-UVM-7-C_{16}, (d) Fe50-UVM-7-C_{16}, (e) Fe50-UVM-7-C_{12}, (f) Fe50-UVM-7-C_{10}, (g) Fe25-UVM-7-C_{16}, (h) and Fe10-UVM-7-C_{16}.

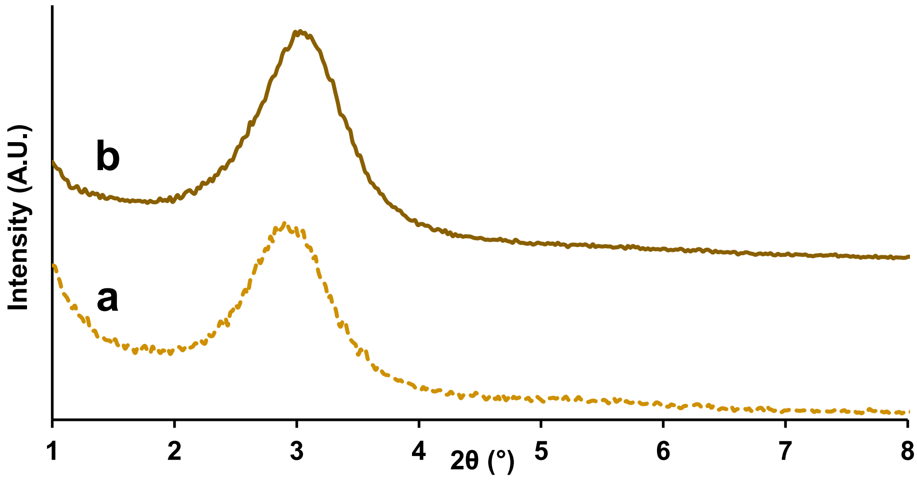

Figure S3. Low-angle XRD patterns of the Fe50-UVM-7-C_{12} material (a) before its use as a sorbent (reference), and (b) after being used 5 times for the extraction of PFASs from water samples.
